# Molecular Basis of Chronic Intestinal Wall Fibrosis in Inflammatory Bowel Diseases

**DOI:** 10.3390/ijms26125754

**Published:** 2025-06-16

**Authors:** Patrycja Sputa-Grzegrzolka, Anna Socha-Banasiak, Piotr Dziegiel, Bartosz Kempisty

**Affiliations:** 1 Division of Anatomy, Department of Human Morphology and Embryology, Faculty of Medicine, Wroclaw Medical University, 50-368 Wroclaw, Poland; 2 Department of Gastroenterology, Allergology and Pediatrics, Polish Mother’s Memorial Hospital-Research Institute, 93-338 Lodz, Poland; 3 Division of Histology and Embryology, Department of Human Morphology and Embryology, Faculty of Medicine, Wroclaw Medical University, 50-368 Wroclaw, Poland

**Keywords:** IBD, Crohn’s disease, ulcerative colitis, EMT, intestinal wall fibrosis, TGF-β, TNF-α

## Abstract

Inflammatory bowel diseases (IBDs), including Crohn’s disease (CD), ulcerative colitis (UC), and IBD-unclassified (IBD-U), are chronic inflammatory disorders of the gastrointestinal tract. Chronic inflammation in the course of IBD is an important initiating factor of fibrosis of the intestinal wall. Intestinal fibrosis is one of the most common and important complications of IBD and, due to the irreversibility of the process and the need for surgical treatment, currently poses a major clinical challenge. In this review, we presented in detail the process of intestinal wall fibrosis at the molecular, immunological, and clinical levels. We characterized the mediators, including transforming growth factor β (TGF-β), tumor necrosis factor-α (TNF-α), and others participating in this process. We also described the type 2 epithelial–mesenchymal transition (EMT) process closely associated with chronic inflammation, leading to excessive development of connective tissue in the intestinal wall in the course of IBD.

## 1. Introduction

Understanding the complex processes leading to intestinal wall fibrosis in the course of inflammatory bowel diseases (IBDs) represents one of the key challenges in contemporary gastroenterology. Elucidating the intracellular molecular mechanisms within enterocytes of the intestinal mucosa, as well as the interactions between epithelial cells, lamina propria fibroblasts, the extracellular matrix, and immune system components, requires a multidisciplinary approach that integrates medicine, molecular biology, and immunology. Only such an integrative strategy can offer novel perspectives for the prevention and treatment of severe IBD-related complications, such as fistulas and strictures, which represent irreversible consequences of chronic inflammation. In this study, we aim to explore the aforementioned processes in greater detail [1,2].

## 2. Inflammatory Bowel Disease (IBD)

IBD is a group of inflammatory diseases of the gastrointestinal tract that includes Crohn’s disease (CD), ulcerative colitis (UC), and the less commonly diagnosed IBD-unclassified (IBD-U) [1].

These diseases are chronic and characterized by alternating periods of exacerbation and remission of symptoms. Interestingly, each patient experiences the disease in a unique way, and the severity and course of the disease depend on the location of the lesions and the amount of inflammation [2,3].

In the general population, the incidence of IBD diagnosis is steadily increasing, which means that the problem continues to pose a serious clinical challenge. Currently, the global prevalence of IBD is estimated at nearly 90 cases/100,000 people. Importantly, the prevalence of the disease is variable in different geographic regions, with the number of new cases increasing rapidly in intensively developing countries. The highest incidence of IBD is observed in Europe, with a rate of 322/100,000 for CD and 505/100,000 for UC [4,5,6].

IBD is a group of diseases diagnosed as early as young adulthood, most often in the third and fourth decades of life for CD diagnosis and in the fourth and fifth decades for UC. Also noteworthy is the proportion of cases reported in the pediatric population, which is about 20–25%, and importantly, among these, as many as 15% are very early-onset IBD (VEO-IBD) [4,6,7]. We use the term VEO-IBD when the onset of the disease affects children before the age of 6. Over the last decade, the incidence of VEO-IBD has been increasing, which means that the age of diagnosed patients is getting younger every year. Clinically, in this group of patients, we usually observe a more severe course of the disease compared to older children and adults and differences in a number of aspects, such as lesion localization, response to treatment, or genetic susceptibility, taking into account rare monogenic disorders, among others. It is worth noting that the onset of the disease at such a young age can affect the child’s subsequent growth and development [8,9].

Despite the many studies carried out, the etiology of IBD has yet to be definitively determined. It is now recognized that the mechanism of the onset and course of IBD is multifactorial. In predisposed individuals, ongoing interactions between genetic, microbial, and environmental factors lead to an excessive and insufficiently inhibited immune system response, causing chronic inflammation in the gastrointestinal wall, resulting in, among other issues, a progressive fibrosis process [10,11].

Patients with any form of IBD exhibit a local inflammatory infiltrate with associated epithelial barrier damage, increased secretion of pro-inflammatory cytokines and oxidative stress, and impaired apoptotic processes [12]. Through the damaged epithelial barrier, bacterial antigens gain access to antigen-presenting cells. We distinguish between the responses of type 1 T-helper cells (Th1 cellular response) and type 2 T-helper cells (Th2 humoral response). In CD, inflammation is mainly associated with stimulation of Th1 cells promoted by IL-12, IL-15, IL-18, IL-21, and IL-23. In contrast, the mechanism of the inflammatory response in UC is less well understood, but it is now believed that the overriding role is played by oversecreted IL-4, which, by stimulating CD4^+^ lymphocytes to differentiate into Th2, causes an increase in IL-13 production [12,13,14]. Th17 lymphocytes may contribute to the pathogenesis of intestinal fibrosis in IBD through excessive secretion of cytokines, including IL-17A, IL-17F, IL-22, and IL-23. These cytokines can stimulate the activation and proliferation of fibroblasts, myofibroblasts, and other connective tissue cells, leading to increased deposition of extracellular matrix (ECM) components, including collagen. Moreover, Th17 cells interact with macrophages and neutrophils, enhancing their activation and potentially stimulating the production of profibrotic growth factors such as TGF-β and platelet-derived growth factor (PDGF), which may further influence fibrotic remodeling in the intestinal tissue [15].

The different subtypes of IBD differ in their clinical presentation, course, treatment, and prognosis (Table 1).

In CD, the inflammatory process begins in the mucosa and gradually involves all layers of the gastrointestinal wall, resulting in its destruction, fibrosis, and the subsequent formation of fistulas and strictures [16]. The inflammatory process can affect any part of the gastrointestinal tract, from the mouth to the rectum, but mainly involves the distal segment of the ileum and colon [17]. The clinical picture of CD is most often dominated by abdominal pain, usually localized in the right lower quadrant; in addition, some patients have diarrhea with blood. The diagnosis of CD should also be considered in the diagnosis of chronic iron deficiency anemia [18]. Chronic inflammation in the gastrointestinal tract also impairs nutrient absorption, which can lead to growth retardation and delayed puberty [19].

In UC, on the other hand, the ongoing inflammatory process involves only the mucosa and submucosa of the rectum and extends proximally through the entire colon in a continuous manner. Based on the degree of involvement of the colon, we distinguish between proctitis, left-sided colitis, and extensive colitis (pancolitis). The main clinical manifestations of UC include rectal bleeding, rectal urgency, and tenesmus [2,20].

The diagnosis of inflammatory bowel diseases (IBD), including Crohn’s disease (CD) and ulcerative colitis (UC), is based on a comprehensive assessment incorporating clinical symptoms, endoscopic findings, histopathological examination, radiological imaging, and laboratory markers. There is no single gold-standard test; rather, diagnosis relies on a multidisciplinary correlation of findings [1,2]. Endoscopic examination reveals patchy, transmural inflammation in CD, characterized by cobblestoning, skip lesions, strictures, and fistulas. Conversely, UC shows continuous mucosal inflammation beginning in the rectum and extending proximally [5]. Histopathologically, CD is marked by transmural lymphoid aggregates, non-caseating granulomas, and focal crypt architectural distortion. UC, on the other hand, is characterized by crypt abscesses, uniform crypt distortion, and inflammation confined to the mucosa [6]. Radiological imaging modalities such as magnetic resonance enterography (MRI) or computed tomography enterography (CT) are utilized to assess bowel wall thickening, strictures, and complications including fistulas and abscesses [7]. Laboratory markers supporting IBD diagnosis include inflammatory markers such as C-reactive protein (CRP) and erythrocyte sedimentation rate (ESR), alongside fecal markers like calprotectin which are useful in differentiating IBD from irritable bowel syndrome (IBS) [8].

Summarizing, patients with IBD, despite continuous progress in gastroenterology, appear to be a highly heterogeneous group in terms of initial clinical presentation, additional test results, treatment course, and its final outcome [17].

## 3. Epithelial–Mesenchymal Transition (EMT)

The epithelial–mesenchymal transition (EMT) is a complex process in which epithelial cells lose their characteristics, acquiring those of mesenchymal cells.

Under the influence of many signals from the external environment, polarized epithelial cells undergo many biochemical changes, as a result of which, we observe drastic changes in their transcriptional and epigenetic profile. This results in the repression of proteins that form intercellular junctions in the epithelium and the reorganization of the cytoskeleton. Epithelial cells also lose their polarity and adopt the phenotype of mesenchymal cells, thus increasing their motility and invasiveness. They are also characterized by reduced susceptibility to apoptosis-inducing signals and the production of ECM components [21,22,23].

Initially, EMT was referred to as epithelial–mesenchymal transformation, which suggested that the process was not reversible [24]. With the observation of the potential for phenotypic plasticity of mesenchymal cells, the term “transformation” was replaced by the term “transition”. Thus, EMT is a reversible process, and mesenchymal cells can convert to epithelial derivatives, i.e., undergo mesenchymal–epithelial transition (MET) at any time [22,25].

In the early 1980s, embryologists described EMT as the precise process responsible for embryonic development [26]. Since then, EMT has been the subject of much research. Today, we know that EMT plays a key role in several developmental processes, such as gastrulation, neural crest development, somite dissociation, and lip and palate fusion [21,27].

Importantly, EMT in the mature organism can occur through both physiological and pathological processes. Physiologically, we observe this process in trauma, where keratinocytes adjacent to the wound acquire the ability to migrate to the site of injury through EMT. Pathological processes, on the other hand, include tumor progression and organ fibrosis. Over the past two decades, research has focused precisely on the role of EMT in pathological processes. Today, it is known that EMT has a role in many chronic fibrotic diseases, such as idiopathic pulmonary fibrosis, cirrhosis, and chronic kidney disease. Fibrosis has also been shown to be associated with many chronic inflammatory and autoimmune diseases, including IBD and rheumatologic conditions such as rheumatoid arthritis, systemic scleroderma, Sjögren’s syndrome, and systemic lupus erythematosus [21,22,23].

The scale of the problem of organ fibrosis is indicated by the fact that the fibrotic process is a common condition for many clinical disorders, accounting for more than 35% of all deaths worldwide [28].

Since EMT can occur in three distinct biological environments (embryonic development, tumorigenesis, and tissue regeneration and inflammation), the functional effects of this process are also different [29]. Because of this distinctiveness, at a conference of experts affiliated with the EMT International Association (TEMTIA) in 2007 in Krakow, Poland, and a year later at Cold Spring Harbor Laboratories, the EMT process was classified into three subtypes [22].

Type 1 EMT is associated with implantation, embryo gastrulation, and organogenesis. It gives rise to the mesoderm and endoderm, as well as mobile neural crest cells. During type 1 EMT, a primary mesenchyme is formed, which can subsequently generate a secondary epithelium through the MET process. Most likely, through later EMT processes, the secondary epithelium generates connective tissue cells, including astrocytes, osteoblasts, chondrocytes, and adipocytes. In type 1 EMT, no fibrotic process is observed [22,30,31].

Type 2 EMT is associated with ongoing inflammation and involves wound healing, tissue regeneration, and organ fibrosis. Unlike type 1 EMT, type 2 lasts longer and can lead to impaired organ function and, ultimately, organ failure through progressive, irreversible fibrosis [22,30].

Type 3 EMT involves tumor cells and their previous genetic and epigenetic modifications, particularly in genes that promote cell adhesion, which promotes the development of cancers. The ongoing process of EMT affects cancer progression, as cancer cells can acquire a malignant phenotype through EMT, gaining the ability to migrate and, consequently, infiltrate and metastasize. Importantly, EMT can involve cancer cells to varying degrees, i.e., some cells will retain many epithelial characteristics, while others will completely acquire a mesenchymal phenotype, expressing mesenchymal markers such as alpha-smooth muscle (α-SMA), ferroptosis suppressor protein 1 (FSP1), vimentin, and desmin [22,30].

Interestingly, according to the literature and clinical trial findings on solid tumors, including colorectal, breast, and ovarian cancers, increased expression levels of typical EMT transcription factors such as SNAIL1 and SNAIL2 have been confirmed. Moreover, the results positively correlate with a worse prognosis for survival or recurrence [32].

Due to the significant role of the fibrosis process in the course of IBD, in our work, we will provide a detailed presentation of the type 2 EMT process occurring in the intestine under the influence of ongoing inflammation in the intestinal wall, while also considering the mediators involved in this process. In addition, we will analyze the process of fibrosis of the intestinal wall at the molecular and clinical levels.

## 4. Type 2 EMT

As mentioned above, type 2 EMT is an inflammation-stimulated subtype of EMT of a potentially reversible nature, primarily associated with wound healing, tissue regeneration, and organ fibrosis. During this process, epithelial cells acquire a mesenchymal phenotype characteristic of fibro- or myofibroblasts, among others. At the molecular level, this means the disappearance of markers responsible for the adhesion and polarization of epithelial cells (proteins such as E-cadherin, cytokeratins, occludin, and tight junction protein 1 (zonula occludens protein 1, ZO-1) and the simultaneous appearance of mesenchymal markers (such as vimentin, FSP1), S-100 family proteins, and α-SMA [30]. The cells in which this process takes place gain the ability to migrate, move, and produce extracellular matrix elements, which is essential during regenerative processes and wound healing.

The factors that induce type 2 EMT include inflammatory mediators such as transforming growth factor α (TGF-α), TGF-β, epidermal growth factor (EGF), fibroblast growth factor (FGF), PDGF, vascular endothelial growth factor (VEGF), interleukin 1 (IL-1), and interleukin 6 (IL-6), which are secreted as a response to tissue damage, causing inflammation [33,34]. These mediators affect epithelial cells, which begin to express the transcription factors SNAIL, SLUG, Zeb-1, Zeb-2 (zinc-finger E-box-binding), TWIST, and numerous miRNA molecules (miR-21, miR-155, miR-31, miR-223, and miR-29a), which inhibit the expression of epithelial markers and stimulate the expression of mesenchymal markers in these cells [35,36].

As an expression of these changes, the induced fibro- and myofibroblasts begin to produce numerous ECM elements, such as collagen types I-VI and XVIII, glycoproteins and proteoglycans (including laminin and periostin), and glycosaminoglycans (e.g., hyaluronic acid). They also produce proteins associated with matrix remodeling, such as extracellular matrix metalloproteinases (MMPs) and tissue inhibitors of metalloproteinases (TIMPs) [33,35,37,38].

With chronic inflammation, as occurs in the intestinal wall altered by IBD, stimulation by inflammatory mediators of epithelial cells occurs continuously. The processes that would ultimately lead to wound healing or regeneration of the damaged organ are ongoing, with the ultimate result being the progressive, excessive formation of fibrous connective tissue in the organ. Through pathological accumulation of ECM components, scar tissue develops over time, and clinically, we observe dysfunction and, ultimately, failure of the fibrotic organ. In the case of the intestine, this can include pathological strictures, fistulas, and perforations [39].

**Figure 1:**
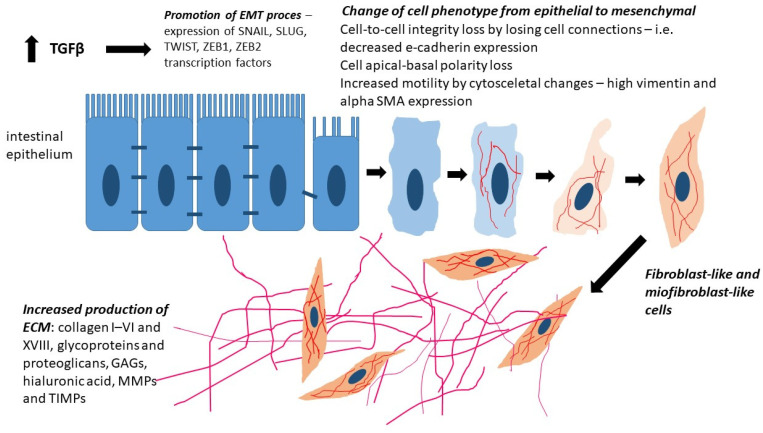
The EMT process is induced by chronic intestinal inflammation and transforming growth factor β (TGFβ) stimulation. EMT drives the expression of transcription factors SNAIL, SLUG, TWIST, ZEB1, and ZEB2, leading to the disruption of intercellular junctions (decreased E-cadherin expression), apical-basal polarity loss, and enhanced cell motility due to cytoskeletal changes (upregulation of vimentin and α-SMA expression). EMT drives epithelial cells to acquire a fibroblast-like and myofibroblast-like phenotype, leading to increased production of ECM components, including collagens (I–VI and XVIII), glycoproteins, proteoglycans, glycosaminoglycans (GAGs), hyaluronic acid, matrix metalloproteinases (MMPs), and their inhibitors (TIMPs).

In conclusion, unlike physiologically occurring type 2 EMT, which gives rise to myofibroblasts from epithelial cells to repair tissue damage, in pathologically chronic inflammation, myofibroblasts induce progressive fibrosis, which leads to the destruction of the organ parenchyma due to excessive ECM deposition [40]. The subsequent stages of EMT in the intestinal wall in IBD are presented in Figure 1.

## 5. Cytokines and Growth Factors Contributing to Intestinal Fibrosis

In susceptible patients, the activation of mesenchymal cells leads to the development of fibrosis instead of orderly wound healing. These processes predominate in the stricturing form of CD. Among the mediators, we distinguish growth factors TGF-β, PDGF, connective tissue growth factor (CTGF), and insulin-like growth factor (IGF), cytokines (IL-1, IL-6, IL-13, IL-17, IL-23, and TNF-α, and others, including reactive oxygen species (ROS), MMPs, and tissue inhibitors of metalloproteinases (TIMPs) [41,42].

### 5.1. Transforming Growth Factor β (TGF-β)

The TGF-β family of proteins includes pleiotropic secreted signaling molecules with potent and unique immunoregulatory properties. There are three TGF-β isoforms, designated TGF-β1, TGF-β2, and TGF-β3. The increased production and activation of TGF-β is observed in immune defects associated with autoimmune diseases, susceptibility to opportunistic infections, and fibrotic complications in diseases with chronic inflammation, including IBD. In the case of fibrosis of the intestinal wall in IBD, it is TGF-β (or, more precisely, mainly its TGF-β1 isoform) that plays an overarching role [6,41,42]. This is confirmed by the studies of Letterio et al. and Li et al. [43,44] on surgically resected stricturing bowel segments, where the expression of the TGF-β1 and TGF-β3 isoforms was higher in smooth muscle cells, myofibroblasts, and fibroblasts located in intestinal strictures compared to sections without strictures in the same patient [41,42]. In a number of studies on scarring during cutaneous wound healing in a rat model, it was observed that TGF-β1 and TGF-β2 are mainly profibrotic, while TGF-β3 promotes wound healing without excessive fibrosis [45,46,47].

TGF-β signaling is mediated by intracellular signal transduction pathways associated with Smad (canonical) and Smad-independent (non-canonical) proteins. In the progression of fibrosis, canonical TGF-β signaling by Smads plays a central role. By binding to the TGF-βR1 and TGF-βR2 receptors, the TGF-β molecule initiates specific intracellular signaling, which is mediated by phosphorylation of Smad 2 and Smad 3 molecules, which bind to Smad 4 to form a complex that translocates to the cell nucleus, regulating genes associated with the EMT process (including affecting the inhibition of E-cadherin expression and stimulating the expression of mesenchymal markers). This process is negatively regulated by Smad 7 [41,43,48].

TGF-β, through a non-canonical pathway (mediated by MAPK, PI3K/AKT, and Wnt/β-catenin), is responsible for the activation of all types of mesenchymal cells found in the intestinal wall, stimulating them to produce ECM components, including type I collagen, which accounts for about 70% of intestinal collagen [6,48,49].

In addition to TGF-β1 promoting the EMT process and endothelial–mesenchymal transition (EndoMT) in intestinal epithelial (and endothelial) cells, which increases the pool of fibroblasts and myofibroblasts, TGF-β1 also stimulates myofibroblast proliferation and immunizes them against pro-apoptotic stimuli [48,50]. Additionally, TGF-β1 also influences ECM remodeling by increasing the expression of tissue inhibitors of metalloproteinases (TIMPs), thereby reducing the MMP/TIMP ratio, which inhibits local ECM degradation and promotes fibrosis [41,50,51,52].

Currently, the differential expression of the three individual TGF-β isoforms by myofibroblasts in IBD is the subject of many studies. McKaig et al. [45] were the first to conduct studies demonstrating significant differences in the secretion profile of the TGF-β isoforms in CD, UC, and healthy intestine. Intestinal myofibroblasts from normal intestinal mucosa predominantly secreted TGF-β3, while in patients with active UC, the expression of TGF-β1 and TGF-β3 was observed. However, myofibroblasts isolated from the mucosa of patients with fibrotic CD secreted increased levels of TGF-β2 and significantly less TGF-β3, and additionally, the proliferation of myofibroblasts in CD was significantly greater than in the other two studied groups. Moreover, TGF-β2 is considered the most effective inhibitor of epithelial proliferation, so its increased expression by intestinal myofibroblasts in CD may be responsible for the persistent epithelial ulceration often observed in CD patients [51].

**Figure 2:**
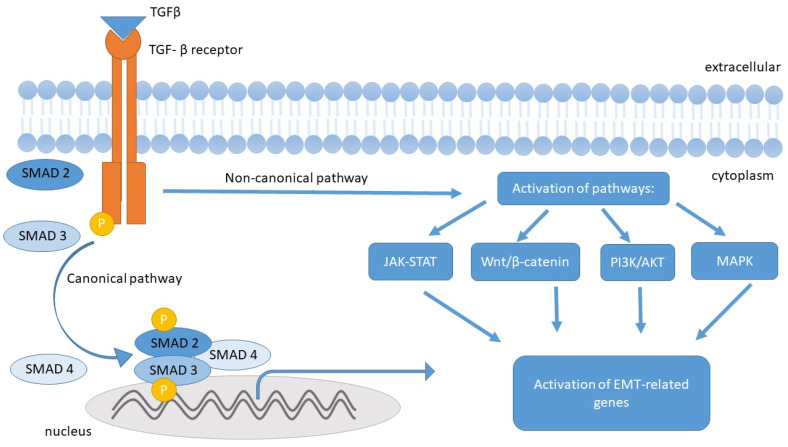
The influence of TGF-β on the induction of the epithelial–mesenchymal transition (EMT) in epithelial cells. Upon TGF-β binding to its receptor, two major signaling pathways are activated: the canonical and non-canonical pathways. In the canonical pathway, SMAD2 and SMAD3 proteins are phosphorylated and subsequently form a complex with SMAD4. This complex translocates to the nucleus, where it regulates the expression of EMT-related genes. The non-canonical pathway activates additional signaling cascades, including JAK-STAT, Wnt/β-catenin, PI3K/AKT, and MAPK, which also contribute to EMT gene activation and promote the phenotypic transformation of cells.

According to Flynn et al., TGF-β1 is particularly increased in the smooth muscle cells of the strictures in the ileum compared to the histologically normal proximal resection margin [53]. The detailed relationship between TGF beta and the EMT process is presented in Figure 2.

### 5.2. Platelet-Derived Growth Factor (PDGF)

PDGF is a dimeric protein composed of two chains: the larger A-chain and the smaller B-chain. PDGF, as a growth factor, stimulates the proliferation of mesenchymal cells and also induces the expression of collagenases secreted by fibroblasts into the ECM [54]. PDGF is secreted by many cells, including smooth muscle cells, endothelial cells, fibroblasts, and activated macrophages [53]. According to several studies, PDGF expression increases in stricturing lesions in CD patients [41,55], but its effect on fibroblasts is not clear. According to the results of some in vitro studies, depending on the dose of PDGF, a decrease in the production of type III collagen is observed [54], while other studies have shown an increase in collagen secretion in the presence of PDGF [41,50,56].

Moreover, PDGF induces the expression of α-SMA in fibroblasts, and increased PDGF activity promotes ECM deposition [57].

### 5.3. Pro-Inflammatory Interleukins IL-1 β, IL-17, and IL-33

Pro-inflammatory cytokines IL-1 β and IL-17 are associated with the pathogenesis of IBD. In the chronic inflammatory process of the intestines, IL-1 β contributes to fibrosis through the activation of myofibroblasts, the secretion of chemokines, and the induction of MMP activity. Moreover, IL-1 β, together with TNF and IFN-γ, enhances TGF-β-induced EMT [41,57]. IL-17, being pro-inflammatory, is a strong activator of mesenchymal cells and promotes the production of a chemokine responsible for the activation of granulocytes [55,56,58]. IL-17A increases the production and secretion of collagen and also induces EMT in a TGF-β1-dependent manner [57].

IL-33, a new member of the IL-1 family, is responsible for mucosal pathology in vivo and may lead to the development of fibrosis and angiogenesis [57]. In a study conducted by Sponheim et al., an increase in IL-33 mRNA levels was observed in patients with UC. The main source of IL-33 in UC lesions was ulcer-associated myofibroblasts, and interestingly, this is a significant difference compared to patients with CD, where IL-33-positive myofibroblasts were almost absent near deep intestinal ulcers [59].

### 5.4. Tumor Necrosis Factor-Alpha (TNF-α)

TNF-α is one of the key pro-inflammatory cytokines associated with the pathogenesis of IBD. It is mainly released by monocytes, lymphocytes, and macrophages [12]. TNF-α plays a critical role in local inflammation [60], and the increase in local TNF-α synthesis triggers a cascade of immunological events through the excessive secretion of pro-inflammatory cytokines (IL-1a, IL-1b, IL-2, IL-6, IL-8, IL-12, IL-17, IL-23, and IFN) and a decrease in the synthesis of anti-inflammatory cytokines (IL-4 and IL-10) [12].

In the clinical aspect, TNF-α inhibitors have been successfully introduced into the therapy of chronic inflammatory diseases. These include monoclonal antibodies directed against TNF-α: infliximab, adalimumab, certolizumab, and golimumab [61]. Anti-TNF-α therapies are established methods of biological treatment for moderate-to-severe forms of CD [62]. The effects of anti-TNF-α treatment are the subject of numerous studies and clinical observations. The effectiveness of anti-TNF-α agents in inducing and maintaining mucosal healing in patients with CD and UC was confirmed by a systematic review with meta-analysis conducted by Cholapranee et al. [63]. However, in a systematic review with meta-analysis prepared by Ford et al., biological treatment was found to be more effective than placebo in inducing remission of active forms of CD and UC, as well as preventing relapses of quiescent CD [64]. Nevertheless, biological treatment does not always yield the desired results—studies indicate that approximately 20% of patients do not respond to anti-TNF-α therapy [65].

It is also worth noting that several other biologic drugs have been approved for clinical use, including integrin inhibitors (vedolizumab and natalizumab), IL-12 and IL-23 antagonists (ustekinumab), and Janus kinase inhibitors (tofacitinib). Despite the growing number of available biological therapies, remission rates in patients with IBD remain unsatisfactory [66]. Therefore, the search for new therapeutic methods for the treatment of IBD based on a deep understanding of the processes involved in its pathogenesis remains a clinical challenge.

## 6. The Fibrosis Process in IBD

In IBD, the chronic inflammatory process leads to the disruption of the epithelial barrier and the destruction of gastrointestinal tissues. Fibrosis, as a mechanism of tissue healing, becomes progressive and harmful in long-term IBD, where persistent tissue damage and healing result in the formation of scar tissue [41,67]. The process of fibrosis is a common effect of the natural course of IBD and serves as the backdrop for most complications, such as strictures, intestinal perforation, and obstruction, which very often require surgical treatment [67,68,69]. For this reason, intestinal fibrosis is a significant clinical problem, constitutes a serious complication of IBD, and affects the further course of the disease and the quality of life of patients.

In patients with UC, the reparative processes occurring in response to inflammation often effectively restore the normal architecture of the intestinal mucosa [41]. The changes are mostly scattered and superficial, and deep ulcers are observed only in patients with severe UC [10]. Strictures in UC are rare, with an estimated 1% to 12% of patients with UC suffering from complications in the form of fibrosis [67,70], and most of them are mild and reversible [71]. However, it is important to note that according to a comprehensive assessment by Gordon et al. in 2018, the progressive fibrosis and thickening of the muscularis mucosae (MM) observed in UC correlate with the severity and chronicity of the inflammation [70]. Therefore, deep remission, including histological remission, should be the main therapeutic goal [72]. Clinically significant is the fact that, with intestinal fibrosis in UC, wall stiffness increases, which can result in abnormal gastrointestinal motility, rectal urgency, and fecal incontinence [67,70,73].

Conversely, in patients with CD, the observed fibrosis is an irreversible condition and causes permanent narrowing and constriction of the intestinal lumen [74]. Among patients with CD with existing strictures, a tendency for strictures to recur is observed [17]. Furthermore, in patients after abscess surgery, fistulas, or peritonitis, the risk of further penetrating complications increases [75]. These observations led to the definition of three types of CD progression: nonpenetrating/nonstricturing (or inflammatory) (B1), penetrating (B2), and stricturing. (B3). Moreover, the CD phenotype in a patient can change over the course of the disease [76,77]. In the early years of the disease, most patients with CD present a purely inflammatory phenotype without structural complications (strictures or fistulas) [17], with only 10% of CD patients showing fibrotic-stricturing changes [78,79]. However, after 40 years, most patients experience complications, with the penetrating form predominating, or, less frequently, the stricturing form. Statistically, it is estimated that over 50% of patients with CD will develop a penetrating or stricturing course [17,41,78], and up to 75% of them will ultimately require surgical treatment [80]. Nevertheless, in operated patients, fibrosis often recurs, particularly at the ileocolonic anastomosis site, which can once again lead to a stricturing form and result in the need for further surgical procedures [81,82]. Statistically, approximately 3–5% of patients with CD undergo surgery each year, and a patient with CD typically undergoes surgery every 15–20 years on average [17].

Interestingly, according to recent prospective studies in Japan, among newly diagnosed CD patients between 2016 and 2020, about 35% had a stricturing or penetrating phenotype at the time of disease diagnosis [83].

The mechanisms by which strictures develop in CD remain unclear. Currently, it is believed that the main cause is the existing intestinal inflammation [80,84,85]. Chronic inflammation is responsible for the activation of myofibroblasts, the main effector cells that produce an excessive amount of ECM, thereby creating a fibrogenic environment [80,86,87,88]. Various factors are responsible for the activation of myofibroblasts, including growth factors and cytokines, primarily transforming growth factor β (TGF-β) as the main regulator of fibrosis, as well as IL-1β, IL-13, IL-17, and IL-33 [41,84,86,88].

As we have already mentioned, increasing evidence indicates the role of EMT in the pathogenesis of intestinal fibrosis in IBD [89].

Several molecules involved in the pathogenesis of intestinal fibrosis, also described in more detail above, have been proposed as potential biomarkers of the process. These include α-SMA, vimentin, fibroblast-specific protein 1 (FSP1), IL-33, periostin, and microRNAs such as miR-21, miR-155, and miR-29a [42,90,91,92]. In addition, TGF-β1, various collagen isoforms (types I, III, V, VI, and XVIII), MMPs, and their endogenous inhibitors (TIMPs) are also recognized as key markers associated with intestinal wall fibrosis [42,91,93]. The expression and activity of these molecules are, in part, regulated by the IL-1β signaling pathway, which facilitates their transcriptional activation and functional engagement in fibrogenic processes [42,93]. This mechanistic link may underlie the observed therapeutic efficacy of IL-1 inhibitors in patients with CD [42,93].

The treatment of patients with CD who have developed fibrosis is currently a significant clinical challenge due to the limited options for effective therapy. In clinical practice, it is important to differentiate the cause of fibrosis in individual segments of the intestine, as this helps to optimize therapeutic management. Fibrotic strictures require endoscopic or surgical intervention, whereas predominantly inflammatory strictures may be a target for anti-inflammatory treatment, mainly TNF-α inhibitors, and are potentially reversible [79,80].

Clinically, CD becomes symptomatic when the changes are extensive or distal, associated with long-term inflammation, or complicated by strictures, abscesses, and fistulas. Importantly, strictures and fistulas can develop asymptomatically over the years. Stricturing and penetrating lesions can coexist in the same person or even in the same segment of the intestine. The symptoms of the disease are closely related to the location of the lesions. Colorectal disease is often multi-symptomatic, whereas changes in the ileum most frequently cause a latent course for several years [17].

Current evidence indicates that once advanced fibrosis has developed—particularly in the stricturing phenotype of CD but also in other forms of inflammatory bowel disease (IBD)—it becomes largely irreversible, often necessitating surgical resection [94,95]. However, early-stage fibrosis across all IBD subtypes may still be amenable to therapeutic modification, especially through targeted interventions that suppress chronic inflammation and inhibit profibrotic signaling cascades [96]. Numerous studies have demonstrated that early and aggressive anti-inflammatory treatment, particularly with TNF-α inhibitors, can mitigate the progression of inflammation-driven fibrosis in IBD [44,97]. These agents, however, exhibit greater efficacy in preventing fibrogenesis than in reversing established collagen deposition, underscoring the importance of timely therapeutic intervention. Initiating therapy prior to irreversible ECM remodeling appears critical for preserving intestinal wall architecture [98]. Recent research has focused on the development of antifibrotic agents that target key molecular pathways implicated in EMT and myofibroblast activation, including inhibitors of TGF-β1, SMAD signaling, and MMPs [42,99]. While promising results have been reported in preclinical models, no antifibrotic agents have yet received regulatory approval for clinical use in IBD [100]. Emerging therapies currently under investigation include TGF-β inhibitors such as fresolimumab, a monoclonal anti-TGF-β antibody that prevents fibroblast activation and extracellular matrix deposition, central events in fibrosis development [101]. Tyrosine kinase inhibitors (TKIs) such as imatinib inhibit signaling pathways like the platelet-derived growth factor receptor (PDGFR), which are involved in fibroblast proliferation and ECM production [102]. Angiotensin II receptor blockers (ARBs), including losartan, have been shown in animal models to reduce TGF-β-mediated fibrosis [103]. Integrin antagonists, such as agents blocking α4β7 integrins like vedolizumab, primarily reduce leukocyte trafficking but may also mitigate fibrosis-associated inflammation [104]. Additionally, matrix metalloproteinase modulators influence ECM turnover and may help restore a balance between ECM deposition and degradation [105]. Broad-spectrum antifibrotic agents such as pirfenidone and nintedanib, approved for idiopathic pulmonary fibrosis, are being investigated for their anti-inflammatory and antifibrotic potential in intestinal fibrosis [106]. Beyond pharmacologic agents, novel therapeutic directions include microRNA-based therapies that regulate fibrotic gene expression and mesenchymal stem cell therapy, which may offer both immunomodulatory and antifibrotic benefits [107,108]. At present, no pharmacologic agents are approved specifically for the treatment of intestinal fibrosis in IBD. Nonetheless, ongoing research continues to elucidate promising molecular targets such as TGF-β signaling, fibroblast activation, and ECM remodeling, representing viable targets for future therapeutic strategies. In conclusion, while advanced fibrosis in IBD remains a therapeutic challenge, early detection and intervention may prevent disease progression. The development of fibrosis-specific therapies, combined with reliable biomarkers for early-stage detection, holds significant promise for altering the natural history of fibrostenotic CD as well as fibrosis-related complications in other forms of IBD. To provide a clearer overview of the current experimental landscape, Table 2 summarizes key in vitro and in vivo findings related to chronic intestinal wall fibrosis in IBD.

## 7. The Role of Gut Microbiota in the Pathogenesis of Intestinal Fibrosis in IBD

In the etiology of intestinal fibrosis in IBD, particularly in Crohn’s disease CD, the role of the gut microbiota cannot be overlooked. The predominance of pathogenic strains, resulting from excessive exposure to antibiotics and industrial chemicals, contributes to the rising incidence of IBD. According to numerous studies, in genetically predisposed patients with IBD, alterations in the composition of the gut microbiota—along with reduced species diversity—play a critical role in shaping a pathological immune response and exacerbating disease activity. In dysbiotic microbiota, during pathological bacterial translocation, generated mediators induce activation of myofibroblasts and stimulate extracellular matrix production, leading to the deposition of fibrinogen and collagen in the intestinal wall [6,109].

An interesting observation was made in a study comparing the microbiota of CD patients with stricturing disease who had undergone ileocecal resection to those of CD patients who did not require surgery. In post-operative patients, a significant and continuously increasing abundance of Enterobacteriaceae was observed, accompanied by a reduction in Parabacteroides and Clostridiales populations [110].

There is also evidence of a direct contribution of specific microbes to the fibrotic process. For instance, in mice infected with enteroinvasive Escherichia coli (EIEC), intestinal fibrosis was observed via a flagellin-dependent mechanism, involving IL-33 induction and activation of the IL-33 receptor [111]. Another microorganism implicated in fibrosis is Salmonella. In mice infected and colonized with Salmonella, not only was intestinal inflammation observed, but also significantly increased expression of TGF-β, connective tissue growth factor (CTGF), and IGF, which collectively contribute to fibrotic remodeling of the intestinal wall [112].

Despite extensive research, the topic of gut microbiota and host–bacteria interactions still requires further investigation. A deeper understanding of these mechanisms may pave the way for more precise and effective therapeutic interventions in the future.

## 8. Conclusions

The progressive process of fibrosis is a significant problem and a clinical challenge, as it initially leads to impaired organ function and, ultimately, to organ failure. This process predominates, among other factors, in chronic, long-lasting inflammatory bowel diseases. The interactions occurring between epithelial cells, immune cells, and fibroblasts play a crucial role in the development and progression of intestinal fibrosis in the course of IBD. Understanding these complex interactions may provide new targets for therapeutic interventions to optimally treat already fibrotic intestines as well as prevent intestinal fibrosis in patients suffering from IBD.

## Figures and Tables

**Table 1:** Differences between Crohn’s disease and ulcerative colitis.

Characteristic	Crohn’s Disease	Ulcerative Colitis
Site of lesions	Segmental changes throughout the gastrointestinal tract, most commonly at the ileocecal junction.	Continuous changes localized in the rectum and extending proximally towards the colon.
Extent of affected structures in the intestinal wall	Changes begin in the mucosa and gradually involve all layers of the intestinal wall.	Changes only in the mucosa and submucosa of the intestine.
Clinical features	Abdominal pain (usually in the right lower quadrant) and bloody diarrhea	Rectal bleeding, rectal urgency, and a feeling of incomplete bowel movement.
Intestinal complications	Fistulas, abscesses, strictures, perforations, obstruction, cachexia, and malabsorption syndrome.	Gastrointestinal bleeding, toxic megacolon, and increased risk of malignancy.
Immunological basis	Stimulation of Th1 cells is promoted by IL-12, IL-15, IL-18, IL-21, and IL-23.	Excessive secretion of IL-4, which stimulates CD4^+^ lymphocytes to differentiate towards Th2 and leads to an increased production of IL-13.

**Table 2:** Key in vitro and in vivo findings on chronic intestinal wall fibrosis in IBD.

Type of Study	Key Findings	Conclusions on Intestinal Fibrosis	Reference
In vitro studies on human intestinal fibroblasts	Overexpression of TGF-β1/β2, decreased TGF-β3; increased proliferation and type I collagen production	TGF-β1 and TGF-β2 promote fibrosis; TGF-β3 may have anti-fibrotic effects	McKaig et al. [45]., Flynn et al. [53]
In vitro: fibroblasts exposed to PDGF	Dose-dependent effect on type III collagen production	PDGF can either stimulate or limit fibrosis depending on context	Stallmach et al. [54], Lawrance et al. [56]
In vitro: IL-17A stimulation of fibroblasts	Increased collagen production and TGF-β1-dependent EMT induction	IL-17A enhances EMT and fibrotic responses	Hata et al. [58]
In vivo: resected bowel segments from CD patients (strictured vs. non-strictured areas)	Higher expression of TGF-β1 and TGF-β3 in fibroblasts, smooth muscle cells, and myofibroblasts	Confirms in vivo role of TGF-β1 in promoting fibrosis	Letterio et al. [43]., Li et al. [44]
Animal model (cutaneous wounds in rats)	TGF-β1/2 promote scarring; TGF-β3 reduces fibrosis	Different TGF-β isoforms have opposing roles—therapeutic potential of TGF-β3	Shah et al. [46]
In vivo: human IBD biopsies	Upregulation of IL-33 in UC lesions, absent in CD	IL-33 may serve as a biomarker distinguishing fibrotic phenotypes in IBD	Sponheim et al. [59]
Clinical observations in CD patients	Most patients develop strictures or fistulas within 40 years of disease	Chronic inflammation drives irreversible fibrosis	Cosnes et al. [76]., Li et al. [44]
In vivo: analysis of SMAD2/3/4 and α-SMA expression	TGF-β activates SMAD and PI3K/AKT pathways; increased α-SMA and type I collagen	TGF-β promotes EMT and myofibroblast proliferation	Yun et al. [48], Ghorbaninejad et al. [49]

## Abbreviations

The following abbreviations are used in this manuscript:

IBD	Inflammatory bowel disease
CD	Crohn’s disease
UC	Ulcerative colitis
IBD-U	IBD-unclassified
TGF-β	transforming growth factor β
TNF-α	tumor necrosis factor-α
EMT	epithelial–mesenchymal transition
VEO-IBD	very early-onset IBD
ECM	extracellular matrix
MET	mesenchymal–epithelial transition
FSP1	ferroptosis suppressor protein 1
α-SMA	alpha-smooth muscle actin
TGF-α	transforming growth factor α
EGF	epidermal growth factor
FGF	fibroblast growth factor
PDGF	platelet-derived growth factor
VEGF	vascular endothelial growth factor
TIEMTA	the EMT International Association
ZO-1	zonula occludens protein 1
Zeb-1	zinc-finger E-box binding protein 1
Zeb-2	zinc-finger E-box binding protein 2
GAGs	Glycosaminoglycans
CTGF	connective tissue growth factor
ROS	reactive oxygen species
MAPK	Mitogen-activated protein kinase
JAK-STAT	Janus kinase—Signal transducer and activator of transcription signaling pathway
PI3K/AKT	Phosphoinositide 3/serine/threonine-specific protein kinases signaling pathway
SMADs	Sma- and Mad-Related Proteins
MMPs	matrix metalloproteinases
TIMPs	tissue inhibitors of metalloproteinases
FSP1	fibroblast-specific protein 1
TKIs	tyrosine kinase inhibitors
PDGFR	platelet-derived growth factor
ARBs	angiotensin II receptor blockers
EIEC	enteroinvasive Escherichia coli
CTGF	connective tissue growth factor
IGF	insulin-like growth factor
IL-1	interleukin 1
IL-1aIL-1bIL-1β	Interleukin 1ainterleukin 1binterleukin 1β
IL-2	interleukin 2
IL-4	interleukin 4
IL-6	interleukin 6
IL-8	interleukin 8
IL-10	interleukin 10
IL-12	interleukin 12
IL-13	interleukin 13
IL-15	interleukin 15
IL-17	interleukin 17
IL-17A	interleukin 17A
IL-17F	interleukin 17F
IL-18	interleukin 18
IL-21	interleukin 21
IL-22	interleukin 22
IL-23	interleukin 23
IL-33	interleukin 33
